# *Pleurotus* Mushrooms Content in Glucans and Ergosterol Assessed by ATR-FTIR Spectroscopy and Multivariate Analysis

**DOI:** 10.3390/foods9040535

**Published:** 2020-04-24

**Authors:** Georgios Bekiaris, Dimitra Tagkouli, Georgios Koutrotsios, Nick Kalogeropoulos, Georgios I. Zervakis

**Affiliations:** 1 Laboratory of General and Agricultural Microbiology, Agricultural University of Athens, 11855 Athens, Greece; giorgosbekiaris@yahoo.gr (G.B.); georgioskoutrotsios@gmail.com (G.K.); 2 Department of Nutrition and Dietetics, School of Health Science and Education, Harokopio University of Athens, 17676 Athens, Greece; d_tagkouli@yahoo.gr (D.T.); nickal@hua.gr (N.K.)

**Keywords:** mushroom, *Pleurotus*, glucan, ergosterol, mid-infrared spectroscopy, FTIR, spectroscopy, chemometrics, prediction

## Abstract

Attenuated total reflectance-Fourier transform infrared (ATR-FTIR) spectroscopy was used to monitor the infrared absorption spectra of 79 mushroom samples from 29 *Pleurotus ostreatus*, *P. eryngii* and *P. nebrodensis* strains cultivated on wheat straw, grape marc and/or by-products of the olive industry. The spectroscopic analysis provided a chemical insight into the mushrooms examined, while qualitative and quantitative differences in regions related to proteins, phenolic compounds and polysaccharides were revealed among the species and substrates studied. Moreover, by using advanced chemometrics, correlations of the recorded mushrooms’ spectra versus their content in glucans and ergosterol, commonly determined through traditional analytical techniques, allowed the development of models predicting such contents with a good predictive power (*R*^2^: 0.80–0.84) and accuracy (low root mean square error, low relative error and representative to the predicted compounds spectral regions used for the calibrations). Findings indicate that FTIR spectroscopy could be exploited as a potential process analytical technology tool in the mushroom industry to characterize mushrooms and to assess their content in bioactive compounds.

## 1. Introduction

During the last two decades, there has been a 30-fold increase in the global supply of cultivated edible mushrooms, following their constantly increasing consumption [1]. *Pleurotus* mushrooms hold the second place in the total production, which is mostly due to their relative ease of cultivation in a wide range of lignocellulosic agro-residues combined with their rather limited infrastructure requirements [1,2,3,4,5]. *P. ostreatus* presents a cosmopolitan distribution and is the most widely cultivated *Pleurotus* species. However, during the last decade, *P. eryngii* (“king oyster”) mushrooms demonstrated a steep increase in demand that is mainly attributed to their excellent organoleptic properties resulting in a 3–5 times higher selling prices in comparison with *P. ostreatus* [1]. Similarly, *P. nebrodensis* is also a choice edible mushroom species and the only fungus included in the Top 50 Mediterranean Island Plants [6]; therefore, its commercialization is of significant importance.

*Pleurotus* mushrooms are of significant nutritional value (i.e., relatively high content in proteins, vitamins and minerals, low amount of fats) which make them ideal for consumption by people suffering from hypertension, high blood low-density lipoprotein (LDL), cholesterol or triglycerides levels, obesity, metabolic diseases and diabetes [5]. Furthermore, they contain bioactive compounds associated with antitumor, antioxidant and immunomodulating activities [7]. Among them, β-glucans are high molecular weight constituents of fungal cell walls found also in *Pleurotus* species, which are linked with health-beneficial properties [8,9,10]. Ergosterol exists in fungal cell membranes and is a well-known precursor of vitamin D_2_ [11]; *Pleurotus* mushrooms, in particular, demonstrate relatively higher concentrations and better conversion kinetics of ergosterol to vitamin D_2_ in respect to other cultivated species [12]. Due to the significance of such bioactive compounds, recent studies focused on increasing their content, and therefore the nutraceutical properties of the mushrooms, by modifying/optimizing the production processes [13,14,15].

*Pleurotus* mushrooms are commonly cultivated on substrates composed of cereal straw supplemented with wheat-, rice- or soy-bran and/or flours from various leguminous seeds. In addition, many other locally abundant plant and food residues are also used for their large-scale production [5]. Among them, olive mill and winery by-products are two of the main agro-industrial wastes generated in the Mediterranean region, and their effective management and safe disposal are particular challenging due to their huge volume, seasonality of production and physicochemical characteristics (e.g., high phenol, lipid and organic acids content, acidic pH) [16,17]. However, both grape marc and olive mill residues contain organic compounds with bioactive properties [18,19]; therefore, their exploitation as substrates for the production of mushrooms with enhanced functionality is much sought-after, but also feasible, as it was recently demonstrated [2,10,13].

Traditional analytical techniques/assays used for measuring bioactive compounds in mushrooms can be laborious, time-consuming and expensive. Process analytical technology (PAT) provides valuable data about chemical processes to be used for monitoring and optimization purposes [20]. In PAT, the combination of appropriate measurement devices with multivariate statistical analysis (chemometrics) creates tools which can rapidly, accurately and usually non-destructively assess the quality, quantity and certain functional properties of various organic compounds [21]. Fourier transform infrared (FTIR) spectroscopy has been an ideal PAT tool for the food industry [22,23,24,25] since it can provide detailed information about the molecular structure of specific compounds of interest. Moreover, when combined with advanced chemometrics, it leads to the prediction of their content in the final product, thus allowing its implementation in the form of an on-line/at-line process analyzer [26]. In the mushroom industry, such a tool (i.e., predicting the content of mushrooms in selected constituents) could be of great interest both for the growers, as a way of promoting a product of high nutraceutical value, as well as for companies processing mushrooms to produce health promoting foods or drugs/cosmetics. To date, FTIR spectroscopy has been mainly applied to identify various filamentous fungi [27,28], to delimit taxa within the genera *Pleurotus*, *Ganoderma* and *Boletus* [29,30,31], to discriminate among mushroom samples of the same species on the basis of geographic origin [32] or to evaluate the post-harvest quality properties in *Agaricus bisporus* mushrooms [33]. To the best of the authors’ knowledge, no PAT tool exists for the assessment of mushroom content in bioactive compounds.

In this study, attenuated total reflectance-Fourier transform infrared (ATR-FTIR) spectroscopy was applied to obtain a chemical insight into the *Pleurotus* mushrooms produced on various substrates, and to develop chemometric tools to accurately determine/predict their content in glucans and ergosterol.

## 2. Materials and Methods

### 2.1. Biological Material

Twenty-nine strains of *P. ostreatus* (#15), *P. eryngii* (#13) and *P. nebrodensis* (#1) were used in this study. All strains were routinely preserved on potato dextrose agar (PDA, Difco; Fischer Scientific, Hampton, NH, USA) and maintained in the Culture Collection of the Agricultural University of Athens, Laboratory of General and Agricultural Microbiology. Each strain was cultivated in up to three substrates, which resulted in a total of 79 samples used for the determination of glucans and ergosterol contents. Results on ergosterol content in the other 30 samples of *P. ostreatus*, *P. eryngii*, *P. nebrodensis* and *P. citrinopileatus*, obtained in previous experiments [13,14], were added to increase the model’s variance during the calibration, achieving, in this way, a better prediction performance and accuracy.

### 2.2. Cultivation of Pleurotus Species

Three substrates, i.e., wheat straw (WS; control), grape marc plus wheat straw (GM; ratio 1:1 *w*/*w*) and two-phase olive mill waste plus olive leaves (OL; ratio 1:1 *w*/*w*) were used for the cultivation of *Pleurotus* strains. The WS substrate was provided by Dirfis Mushrooms IKE (Kathenoi, Euboea, Greece), grape marc was obtained from a winery in the Nemea area (northeast Peloponnese, Greece), and the two-phase olive-mill wastes and olive leaves from an olive-oil mill situated in Kalamata (southwest Peloponnese, Greece), respectively. Substrates were milled to a particle size of 2−3 cm and soaked in water for 24 h. Water surplus was drained off (moisture content of the substrates was 53%–69%), and the substrates were mixed with calcium carbonate and wheat bran (2% *w*/*w* and 5% *w*/*w*, respectively). Two kg of each formulated substrate was then placed into autoclavable polypropylene bags and sterilized twice for 1 h (121 °C, 1.1 atm). Inoculation of substrates was performed with a spawn (5% *w*/*w*) prepared as described by Koutrotsios et al. [3]. Four replicates per substrate were used. Incubation of cultures and fructification were carried out in a specially-designed mushroom cultivation room under conditions previously reported [3]. Prior to the analyses, the collected mushroom samples were freeze-dried and grinded to a particle size less than 2 mm.

### 2.3. Determination of Glucan and Ergosterol Content

The determination of the mushrooms’ total and α-glucans content was performed by the Mushroom and Yeast Beta-Glucan assay kit (Megazyme Int., Bray, Ireland) according to the manufacturer’s instructions, while the β-glucans content was calculated by subtracting α-glucans from the total glucans. Light absorbance was measured at 510 nm using a Hitachi U-2001 spectrophotometer (Hitachi High-Tech America, Inc.; Schaumburg, IL, USA).

The mushrooms’ ergosterol content was determined as described by Sapozhnikova et al. [34]. Cholesterol (100 μg/mL, internal standard) was added in 100–200 mg of the freeze-dried mushroom sample and saponified with 2 mL of potassium hydroxide (3M) in methanol under sonication (10 min) and heating (60 °C, 60 min). All manipulations were performed under reduced light conditions to avoid the potential conversion of ergosterol to vitamin D_2_. The un-saponified fraction was extracted twice with 3 mL of hexane. Hexane extracts were then pooled and evaporated to dryness (Speed Vac, Labconco Corporation, Kansas City, MO, USA). Sterols were derivatized to trimethylsilylethers (TMS) with N,O-Bis(trimethylsilyl)trifluoroacetamide (BSTFA) at 70 °C for 20 min, and 1 μL aliquots were injected in the gas chromatographer (Agilent HP GC 6890 N; Wallborn, Germany) coupled with a mass spectrometer (Agilent HP 5973; Wallborn, Germany) at a split ratio of 5:1. The analysis of the TMS sterol derivatives was carried out under electron impact ionization (70 eV) and separated by an Agilent J&W HP-5ms capillary column (30 m × 0.25 mm × 250 μm) with a carrier gas flow rate equal to 0.6 mL/min (high-purity He). The injector and MS detector transfer line were kept at 220 °C and 300 °C. The oven temperature was set initially at 210 °C, raised to 300 °C at 5.5 °C/min, and held for 14 min. The identity of ergosterol was verified by the presence of expected ion fragments at the proper ratios according to literature [35,36]. Ergosterol quantification was performed by constructing a 6-point calibration curve, covering the range 0–600 μg, and by employing cholesterol as an internal standard.

### 2.4. Attenuated Total Reflection—Fourier Transform Infrared (ATR-FTIR) Analysis

ATR-FTIR spectra of the mushroom samples were obtained by a Perkin Elmer Spectrum-Two spectrometer equipped with a Diamond ATR compartment (Perkin Elmer, Hopkinton, MA, USA) using the Spectrum 10 software provided by the manufacturer. For each sample, 32 scans of the infrared region between 4000 and 400 cm^−1^ at a resolution of 4 cm^−1^ were recorded in triplicates and averaged. The recorded spectra were then ATR-corrected with a refractive index for diamond of 1.5 in order to be comparable to the available spectral libraries for facilitating the interpretation of spectra.

A spectroscopic analysis followed to obtain a comparative insight among the mushrooms produced by the different *Pleurotus* species on various substrates. Prior to the spectroscopic analysis, the spectra were smoothed by the Savitzky–Golay algorithm (5 points each side (total window of 11 smoothing points) and a zero order polynomial) [37], linear baseline corrected and then normalized by the mean using The Unscrambler X v.10.5 software (CAMO software, Oslo, Norway).

### 2.5. Multivariate Analysis

A principal component analysis (PCA) on the smoothed, baseline-corrected and normalized ATR-FTIR spectra of the mushrooms was performed to detect any grouping in terms of species or cultivation substrate by using The Unscrambler X v.10.5 software (CAMO software, Oslo, Norway). For this purpose, singular value decomposition (SVD) was applied for 20 principal components using a leave-one-out cross-validation. Partial least square regression (PLSR) analysis was performed to calibrate models predicting the glucans and ergosterol contents of mushrooms on the basis of their recorded ATR-FTIR spectra. A wide range of spectral transformations and various combinations were applied to the recorded spectra (i.e., Savitzky–Golay smoothing, smoothing by the median, linear and non-linear baseline correction, normalization by the mean, multiplicative scatter correction, standard normal variate, de-trending, first and second derivative, etc.) to obtain better predictions. Potential sample outliers were detected using the interquartile ranges approach [38] for the measured glucans and ergosterol values, while spectral outliers were identified by Hotelling’s T^2^ distribution [39]. In order to avoid overestimations in predictions, the sample sets for each model (i.e., 79 samples for the glucans prediction and 109 samples for the ergosterol prediction) were divided into a calibration (CAL) set containing nine tenths of the samples and an external validation (EV) set with the remaining samples. The CAL set was used to develop the calibration model on which the optimal number of components was chosen based on a leave-one-out cross-validation (CV; models’ self-testing). The EV set was constructed by selecting every tenth sample following the order of the glucans or ergosterol contents in the mushrooms, and used for the evaluation of the robustness of the developed models. Non-significant variables were removed in some cases by the Martens’ uncertainty test [40] to improve the models’ stability and robustness. The Unscrambler X v.10.5 software (CAMO software, Oslo, Norway) was used for all calibrations.

The models’ performance was determined by the *R^2^* (coefficient of determination) value (Equation (1)):

(1) $$R^{2}=\frac{\Sigma_{i}{\left(y_{i}-f_{i}\right)}^{2}}{\Sigma_{i}{\left(y_{i}-\bar{y}\right)}^{2}}$$

where *yi* represents the measured values and *fi* represents the predicted values. The closer *R^2^* is to 1, the better the fit of the measured values (*yi*) to the regression line.

The models’ precision was determined by the root mean square error (*RMSE*) in % of the dry weight (dw) for the glucans content and mg g^−1^ (dw) for the ergosterol content (Equation (2)):

(2) $$RMSE=\sqrt{\frac{\Sigma_{i=0}^{n}{\left(f_{i}-y_{i}\right)}^{2}}{n}}$$

where *yi* represents the measured values and *fi* represents the predicted values.

In addition, the relative error of prediction (*REP*) given in % [41] was calculated by (Equation (3))

(3) $$REP=100\frac{RMSE}{z}$$

where *z* is the mean value of the calibration concentrations for the analyte examined.

## 3. Results and Discussion

### 3.1. Glucan and Ergosterol Contents of Pleurotus Species

The total glucan content of the *P. ostreatus* strains ranged from 38.84% to 58.90% of dry weight (dw) for the mushrooms produced on the WS substrate, from 28.28% to 48.42% dw for the GM substrate, and from 15.53% to 41.16% dw for the OL substrate. The values range for the β-glucan contents (total glucans minus α-glucans) was 30.18%–48.16% dw (WS), 22.66%–40.56% dw (GM) and 14.62%–31.31% dw (OL). As regards the ergosterol content, the values range measured for the *P. ostreatus* mushrooms was 6.42–16.06 mg g^−1^ dw (WS), 10.94–26.09 mg g^−1^ dw (GM) and 11.82–20.25 mg g^−1^ dw (OL). For *P. eryngii*, the respective contents varied less than in *P. ostreatus*, i.e., the total and β-glucans contents for the mushrooms deriving from the WS substrate were 32.84%–61.40% dw and 26.44%–51.36% dw, respectively, from the GM substrate were 37.08%–54.11% dw and 32.18%–44.73% dw, respectively, while from the OL substrate they were 31.02%–52.69% dw and 27.54%–42.33% dw, respectively. A similar pattern was also observed for the *P. eryngii* strains in respect to the mushrooms’ ergosterol content, which was 4.83–14.26 mg g^−1^ dw (WS), 7.30–14.10 mg g^−1^ dw (GM) and 9.27–19.42 mg g^−1^ dw (OL). As regards the *P. nebrodensis* mushrooms grown on WS and GM, the total glucans were 38.72% and 44.86% dw, while the β-glucans were 30.23% and 35.11% dw, respectively. The ergosterol content was 13.91 mg g^−1^ dw and 12.43 mg g^−1^ dw in the *P. nebrodensis* mushrooms from WS and GM, respectively.

**Figure 1:**
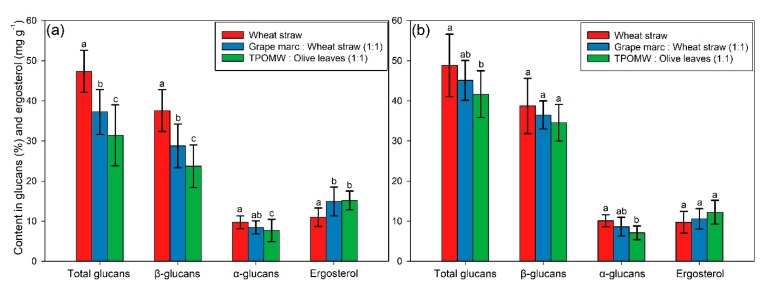
Mushroom contents in the total, α- and β-glucans (% dw), and ergosterol (mg g^−1^ dw) for (**a**) *Pleurotus ostreatus* and (**b**) *P. eryngii* cultivated in three substrates, i.e., wheat straw (control; WS), grape marc plus wheat straw (1:1 *w*/*w*; GM) and two-phase olive mill waste (TPOMW) plus olive leaves (1:1 *w*/*w*; OL). Error bars represent standard deviation among the strains of each species. Lack of letters in common indicates statistically significant differences (Duncan’s *t*-Test, *p* < 0.05) in comparisons of bioactive compounds content between different substrates for each species examined.

The mean of the measured total, α-, β-glucan and ergosterol contents of the *Pleurotus* strains was projected for *P. ostreatus* and *P. eryngii* (Figure 1) in order to obtain a generalized perspective of the effect that different cultivation substrates have on the content of bioactive compounds. The *P. ostreatus* strains revealed a significant decrease in terms of the total and β-glucan contents when substrates other than WS were used (i.e., WS > GM > OL), whereas a significant increase was observed for the ergosterol content in GM and OL (Figure 1a). A similar pattern was observed for the *P. eryngii* strains as regards both glucans and ergosterol (Figure 1b). However, no significant differences between the cultivation substrates were observed for β-glucans and ergosterol as it was the case in *P. ostreatus*, which might be indicative of a reduced impact that the cultivation media could exert on the *P. eryngii* mushroom content in these compounds.

### 3.2. Qualitative Analysis of Pleurotus Mushrooms Based on ATR-FTIR Spectroscopy

#### 3.2.1. Spectral Comparison of *P. ostreatus* and *P. eryngii* Mushrooms Cultivated on Different Substrates

**Figure 2:**
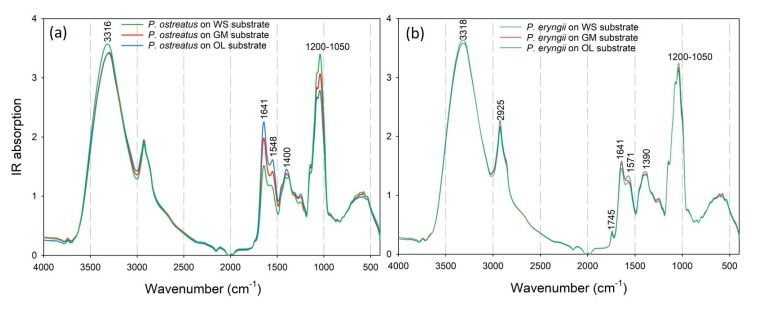
Recorded attenuated total reflectance (ATR)-FTIR spectra of (**a**) *Pleurotus ostreatus* and (**b**) *P. eryngii* mushrooms cultivated on wheat straw (WS), wheat straw plus grape marc mix, 1:1 *w*/*w* (GM) and two-phase olive mill waste plus olive leaves, 1:1 *w*/*w* (OL) substrates.

In order to perform a comparative evaluation among the mushrooms produced on different substrates, the recorded spectra of the *P. ostreatus* strains on each substrate were averaged (Figure 2a). Differences were observed in the IR absorption regions at 3316, 1641, 1548, 1400 and 1200–1050 cm^−1^. Similar peaks were also detected for the *P. eryngii* mushrooms, while an additional peak was evident at 1745 cm^−1^ (Figure 2b). The peak at 3316 cm^−1^ (observed in mushrooms produced in WS, and shifted to 3313 and 3301 cm^−1^ for GM and OL, respectively) can be attributed to the N-H stretching vibration of the amide A band in proteins and nucleic acids or the O-H stretching vibration in phenols and H_2_O [42,43]. The IR absorption peak at 1745 cm^−1^, which was only observed in the spectra of the *P. eryngii* mushrooms, corresponds to the C=O stretching of phospholipids. A similar pattern (i.e., presence in *P. eryngii*, absence in *P. ostreatus*) was also reported by Zervakis et al. [29].

The peak at 1641 cm^−1^ could be associated with the C=O stretching vibration in the amide I band [42], the C=C and C=O stretching vibrations in amino acids [42,43], the N-H bending in flavonoids [43] and the aromatic ring deformations [44]. This specific peak was found to be well correlated with the antioxidant capacity of the propolis samples [45]; therefore, the higher absorption intensity for mushrooms produced on OL and GM is indicative of a higher antioxidant activity of the *Pleurotus* mushrooms deriving from these particular substrates, as previously evidenced [13]. Additionally, the higher absorption intensity in this region for mushrooms cultivated on OL, followed by those originating from GM and WS, is in agreement with their total phenolic content and concurs with previous pertinent findings [13]. The same also applies for the *P. eryngii* mushrooms; however, smaller differences were detected in the IR absorption signal for this peak since the effect of the cultivation substrate on the total phenolic content is not as pronounced as in *P. ostreatus*. The peak around 1550 cm^−1^ could be assigned to the N-H bending vibration and C-N stretching vibration of the amide II region in proteins, while the peak around 1400 cm^−1^ can be associated with the symmetric stretching vibration of the COO- group of fatty acids and amino acids, the symmetric bending modes of methyl groups in skeletal proteins and the symmetric stretch of methyl groups in proteins [42]. The last two peaks (i.e., at 1550 cm^−1^ and 1400 cm^−1^) could indicate a higher protein content in the mushrooms produced on the OL and GM substrates.

Koutrotsios et al. [3] reported an increase in the total crude protein content of the *P. ostreatus* mushrooms cultivated on the OL substrate. The region at 1200–900 cm^−1^ could be assigned to the C-O stretching vibration of the pyranose compounds in carbohydrates [42,44], with the absorption in this region to be more intense for the mushrooms produced on WS (followed by GM and OL), indicating a higher content in polysaccharides. This is in agreement with the glucans content measured in the respective samples (Figure 1a). The differences in this particular region are rather low in the *P. eryngii* mushrooms; however, they are in accordance with their glucans content (Figure 1b), as in the case of *P. ostreatus*.

#### 3.2.2. Comparative Evaluation of Pleurotus Species

**Figure 3:**
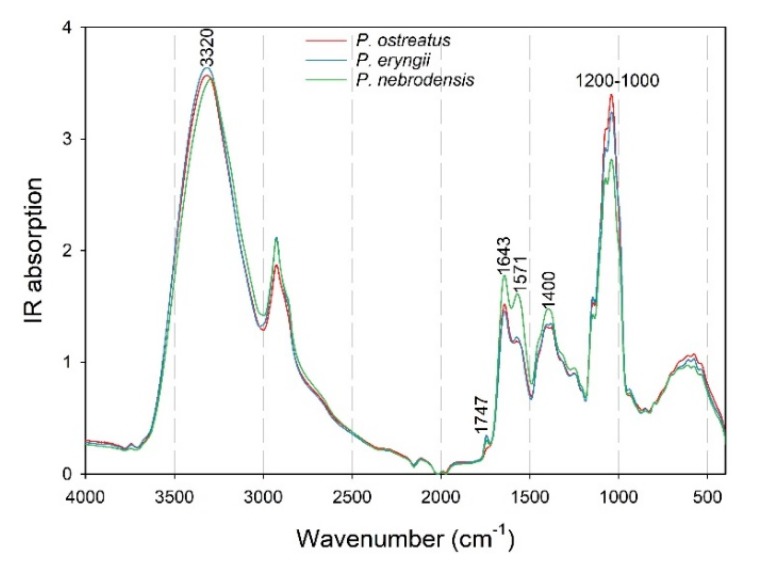
Recorded ATR-FTIR spectra for the *Pleurotus ostreatus*, *P. eryngii* and *P. nebrodensis* mushrooms cultivated on wheat straw (WS).

A spectroscopic comparison of the *P. ostreatus*, *P. eryngii* and *P. nebrodensis* mushrooms cultivated on WS was performed to detect potential spectroscopic differences among the species examined (Figure 3).

Indeed, differences were revealed in the spectral regions at 3320, 1747, 1643, 1571, 1400 and 1200–1000 cm^−1^, which were particularly obvious in the case of *P. nebrodensis*. The peak at 1747 cm^−1^ was evident only in the *P. eryngii* and *P. nebrodensis* spectra, and could be attributed to the C=O stretching of phospholipids. The fact that this peak was produced by these two species only (and not by *P. ostreatus*) is in agreement to their close phylogenetic affinity [46]. The peaks at 1643 cm^−1^ (C=O stretching in the amide I region, C=C and C=O stretching in amino acids, N-H bending in flavonoids and aromatic ring deformations), 1571 cm^−1^ (N-H bending and C-N stretching in the amide II region in proteins) and 1400 cm^−1^ (COO- group symmetric stretching in fatty acids and amino acids, methyl groups symmetric bending in skeletal proteins and methyl group symmetric stretch in proteins) had an increased IR absorption for *P. nebrodensis*, which could indicate an increased protein content of this particular strain in respect to the *P. ostreatus* and *P. eryngii* material. Finally, the region at 1200–1000 cm^−1^, which is related to the C-O stretching vibration of the pyranose compounds in carbohydrates, revealed an increasing IR absorption intensity from *P. nebrodensis* to *P. eryngii* to *P. ostreatus*, in accordance with the total glucans content determined for these particular species.

### 3.3. Principal Component Analysis (PCA)

**Figure 4:**
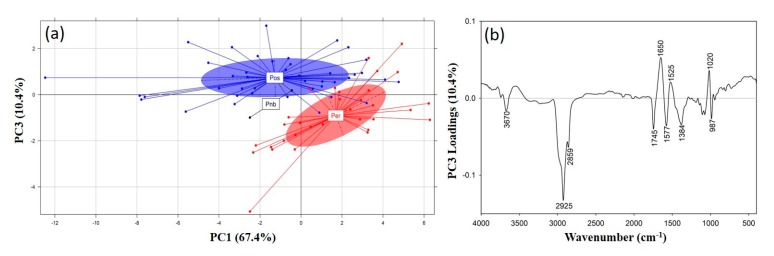
(**a**) Score plot of principal component analysis (PCA) (PC1 vs. PC3) for the discrimination of the *Pleurotus* species (Pos: *P. ostreatus*; Per: *P. eryngii*; Pnb: *P. nebrodensis*) on the basis of their recorded ATR-FTIR spectra, and (**b**) PCA loadings for the PC3 across which discrimination is evident.

A PCA was performed on the ATR-FTIR spectra of the *P. ostreatus*, *P. eryngii* and *P. nebrodensis* mushrooms to detect groupings/associations of interest. Most of the spectral variance (>99%) was explained through the first ten principal components (PCs), with the first three explaining 91% of the variance (i.e., PC1: 67.4%; PC2: 13.2%; PC3: 10.4%). The correlation of the first (PC1) and third principal components (PC3) revealed a fairly clear separation of the *Pleurotus* species on the basis of the recorded spectra (Figure 4a), which is mostly evident across the *y*-axis (PC3).

The PC3 loadings (Figure 4b) were interpreted to identify the spectral regions responsible for this separation, and subsequently to identify potentially related compounds. The *P. eryngii* and *P. nebrodensis* mushrooms exhibited similar spectroscopic characteristics and positively correlated with regions at 2925 and 2859 cm^−1^ (aliphatic compounds), 1745 cm^−1^ (phospholipids), 1577 cm^−1^ (amide II region) and 1384 cm^−1^ (C-N stretching in tertiary aromatic amines; CH_3_ symmetric vibrations in lipids).

On the other hand, the *P. ostreatus* mushrooms were positively correlated with regions at 1650 cm^−1^ (N-H bending of primary amines and C=O stretching in the amide I region) [42,44,47], 1525 cm^−1^ (amide II region) and 1020 cm^−1^ (pyranose compounds in carbohydrates). The latter region is related to the polysaccharide content of mushrooms and is indicative of the higher glucans content in *P. ostreatus*, while the region at 1745 cm^−1^, which was positively correlated to *P. eryngii* and *P. nebrodensis*, was also found to characterize these two species (Figure 3).

**Figure 5:**
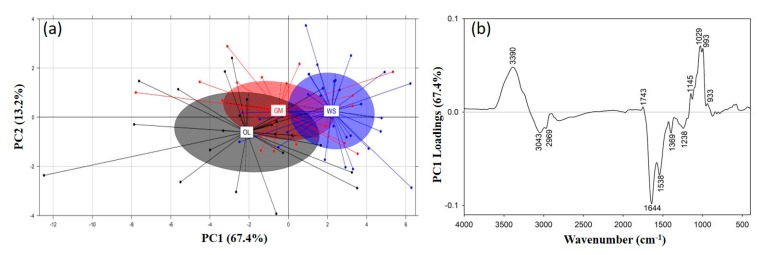
(**a**) Score plot of PCA (PC1 vs. PC2) for the discrimination of substrates (WS: wheat straw; GM: grape marc plus wheat straw, 1:1 *w*/*w*; OL: two-phase olive mill waste plus olive leaves, 1:1 *w*/*w*) on which the *Pleurotus* mushrooms were produced on the basis of their recorded ATR-FTIR spectra, and (**b**) PCA loadings for the PC1 across of which the discrimination is evident.

Furthermore, a PCA was performed for discriminating the mushrooms on the basis of the substrate on which they were produced. Even if discrimination was not so clear, as it was among species, probably due to the similar characteristic compounds present in the mushrooms, the different IR absorption intensities allowed a partial separation, especially of the mushrooms produced on WS and OL through the correlation of the first two PCs (PC1 vs. PC2) (Figure 5a). An interpretation of the PC1 loadings (Figure 5b), across which discrimination was mostly evident, revealed a dominant positive correlation (i.e., the group positioned to the right, positive numbers of this axis) with the region 1200–950 cm^−1^ (polysaccharide region). This fact, combined with the previously reported higher content in glucans for the mushrooms cultivated on WS (Figure 1), confirms the accuracy of this separation.

### 3.4. Prediction of Mushrooms Glucans Content

**Figure 6:**
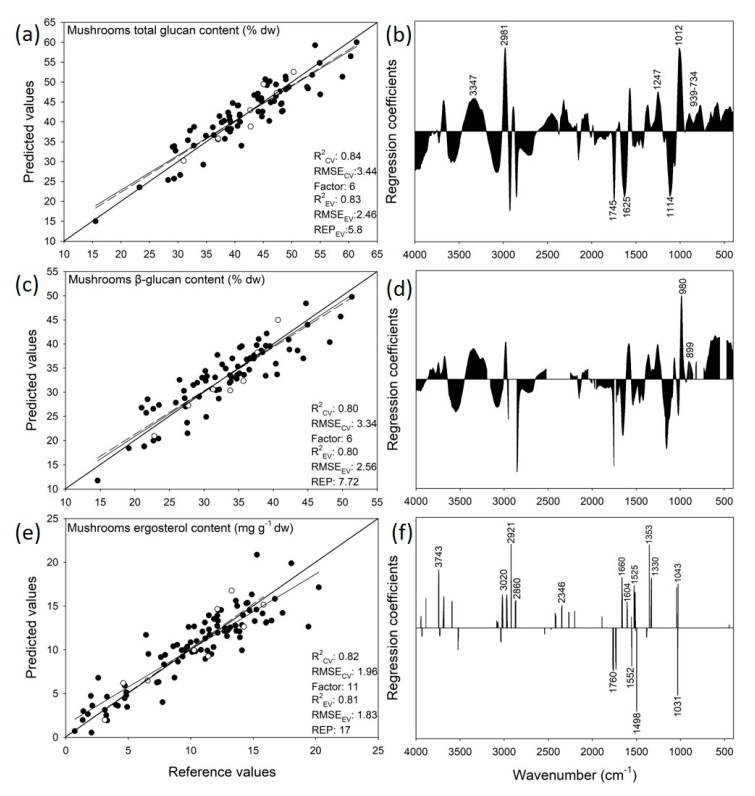
The measured vs. the predicted values and regression coefficients of calibration for the prediction model of (**a**,**b**) the total glucans content, (**c**,**d**) the β-glucans content and (**e**,**f**) the ergosterol content in the *Pleurotus* mushrooms.

PLSR models were developed for the prediction of the total and β-glucans contents of the mushrooms on the basis of their ATR-FTIR spectra. Among various spectral transformations performed prior to the calibration, Savitzky–Golay smoothing (window of 11 smoothing points, zero polynomial) combined with lineal baseline correction and normalization by the mean provided the best precision and accuracy of the calibrated model. The latter, predicting the total glucans content in the *Pleurotus* mushrooms, was developed from a set of 72 samples, leaving out seven samples to be used for the external validation of the model’s performance, accuracy and robustness. A sample was identified as an outlier based on the interquartile ranges approach as well as on the observation of the score plot of the reference vs. the predicted values [48]. For six factors, the developed model achieved an R^2^ value of 0.90 and 0.84 for the calibration (R^2^_CAL_) and cross-validation (R^2^_CV_), respectively, with root mean square error values of 2.77% (RMSE_CAL_) and 3.44% (RMSE_CV_) (Figure 6a). The respective values for the external validation sample set were 0.83 (R^2^_EV_) and 2.46% (RMSE_EV_). Based on that, the relative error of prediction for the external validation (REP_EV_) was calculated at 5.8%, which has been previously characterized as a highly acceptable error value for calibrated models [49].

The developed model predicting the β-glucan content (Figure 6b) was also calibrated for 72 samples (seven samples left out to be used for the external validation), while the same sample set was also identified as an outlier on the basis of the score plot of the reference vs. the predicted values and Hotelling’s T^2^ distribution [39,48]. A removal of non-significant variables was applied using the jack-knifing algorithm [40]. For six factors, the developed model achieved an R^2^ value of 0.86 for the calibration (R^2^_CAL_) and 0.80 for cross-validation (R^2^_CV_), while the root mean square values were 2.73% and 3.34%, respectively. For the external validation sample set, the achieved R^2^_EV_ value was 0.80 with a RMSE_EV_ of 2.56%. Furthermore, the calculated relative error of prediction for the external validation predictions was 7.72%, pointing to a reasonable/acceptable error for this calibrated model.

In order to increase the model’s robustness and regression coefficients (i.e., the spectral regions automatically selected by The Unscrambler software for the calibrations and correlated positively or negatively with the developed prediction models) were interpreted, as a way of eliminating as much as possible the possibility of an artifact (i.e., model calibrated based on irrelevance to the predicted value spectral regions). The interpretation of the regression coefficients revealed that the prediction of the mushrooms’ total glucans content (Figure 6b) was significantly positively correlated with the spectral regions at 3347 cm^−1^ (OH symmetric and asymmetric stretching), 2981 cm^−1^ (methyl group C-H stretching), 1247 cm^−1^ (among others C-H stretching and O-H deformations in carbohydrates), 1012 cm^−1^ (C-O stretching in carbohydrates) and 939–734 cm^−1^ (C-H vibrations related to the α- and β- pyranose compounds, both glycosidic and non-glycosidic) [44]. The former region (i.e., at 3347 cm^−1^) may also refer to the OH groups which are contained at a significant number in the backbone of glucans [50].

On the other hand, a significant negative correlation of the predicted total glucans content could be observed at the regions of 1745 cm^−1^ (C=O in phospholipids), 1625 cm^−1^ (C=O stretching in the amide I band) and 1114 cm^−1^ (C-O stretching in crystalline cellulose) [42,51]. The interpretation of the regression coefficients used for the prediction of the β-glucans (Figure 6d) revealed very similar correlations with the regression coefficients for the total glucans predictions, but in this case the polysaccharide region (between 1200 and 800 cm^−1^) has been replaced by a very strong positive correlation at 980 cm^−1^ and by a positive correlation at 899 cm^−1^. Socrates [44] assigned these regions to the symmetric ring vibration and the C-H deformation, and designated them as characteristic of the β-pyranose compounds.

Unfortunately, due to lack of studies related to the prediction of the total and β-glucans in the mushrooms on the basis of the IR spectroscopic data, a direct comparison of the model developed in this study was not feasible. Ma et al. [52] used near infrared spectroscopy (NIRS) to predict the polysaccharide content in mycelia of *Ganoderma* species and achieved an R^2^_CV_ value of 0.98. Nevertheless, this model was developed for fungi phylogenetically well-separated from *Pleurotus*; more importantly, it was based on the outcome of the analysis of mycelia (and not of fruitbodies) for which the compositional properties can be different [53]. In addition, a different spectroscopic technique was adopted (i.e., NIRS), which reflects mid-infrared overtone regions and combination bands that can be highly overlapping [54]. In this way, very limited information is obtained in respect to the chemical components associated with the regions used for the calibration; hence, a direct identification of the compounds is difficult [55]. Finally, the prediction of the total polysaccharides content makes the model less specific since it includes a wide range of sugar compounds. In a relevant study, Chen et al. [53] reported R^2^ values of 0.973–0.989 and an RMSE_P_ = 0.225–0.012 for a model developed on the basis of NIR spectra of polysaccharides and triterpenoids in *Ganoderma lucidum* and *G. atrum* mushrooms. When the plant material was examined, Gracia et al. [56] obtained a high prediction performance (R^2^ values) of the β-glucan content using NIRS for a sample set of 1728 single intact groats of six different oat varieties, while Brown et al. [57] predicted the total glucans content in *Setaria viridis* plants by using ATR-FTIR with *R*^2^ values of 0.90 for a sample set of 183 collections. In addition, Li et al. [58] used FTIR for the prediction of the total polysaccharides content in Chinese ginseng (*Panax notoginseng*), achieving an R^2^ value of 0.83 for the external validation sample set with relatively a low REP. Although the outcome of these studies is not directly comparable to the model developed in the present work, since different materials were examined, they are indicative of its good predictive power. It is noteworthy that as far as mushrooms are concerned, FTIR and NIR spectra have been also used to build prediction models suitable for addressing other issues, e.g., the post-harvest quality deterioration of *A. bisporus* fruitbodies [33,59] and the geographical traceability of *Boletus* spp. [60,61].

### 3.5. Prediction of Mushrooms Ergosterol Content

The PLSR models predicting the mushrooms’ content in ergosterol were developed on the previously transformed spectra by detrend (second polynomial) and first Savitzky–Golay derivation (window of 11 smoothing points and second polynomial). The calibrated model predicting the ergosterol content in the *Pleurotus* mushrooms was developed on a set of 100 samples, leaving out nine samples for the external validation of the model’s performance, accuracy and robustness. Ten samples from the calibration sample set were identified as the outliers on the basis of the interquartile ranges approach as well as on observations of the score plot of the reference vs. the predicted values and the Hotelling’s T^2^ distribution [39,48]. A removal of non-significant variables was also applied by using the jack-knifing algorithm of Martens and Martens [40], provided by The Unscrambler software. For 11 factors, the developed model achieved an R^2^_CAL_ value of 0.90 and an R^2^_CV_ of 0.82, while the root mean square values were 1.47 mg g^−1^ dw and 1.96 mg g^−1^ dw, respectively. For the external validation sample set, the achieved R^2^_EV_ and RMSE_EV_ values were 0.81 and 1.83 mg g^−1^ dw, respectively (Figure 6e). However, the calculated relative error of prediction for the external validation prediction was approximately 17%, which makes the enhancement of the model’s predictive accuracy necessary, even if it reveals a relative reasonable error. This can be potentially achieved by increasing the number of samples included during calibration.

**Figure 7:**
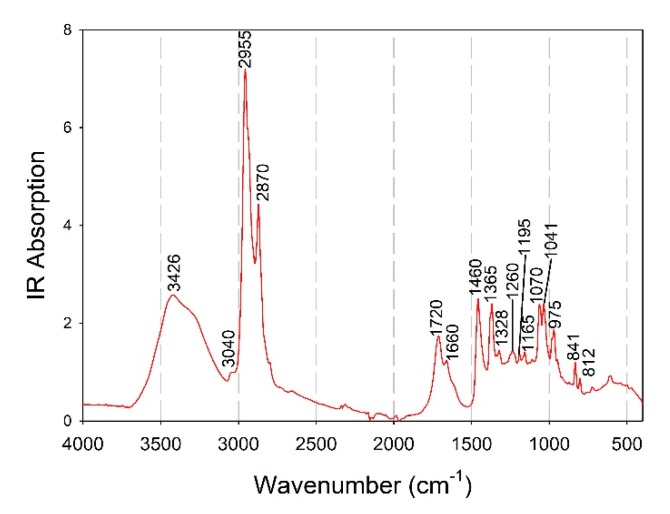
ATR-FTIR spectrum of the ergosterol standard with marked characteristic peaks.

The interpretation of the regression coefficients used during calibration (Figure 6f) would significantly improve the trustfulness of this model, if related ergosterol spectral regions could be used. To address this issue, the spectrum of ergosterol standard compound (Sigma-Aldrich, EC Number 200-352-7, CAS Number 57-87-4) was recorded by using the same spectroscopic setup, and peaks characteristic of ergosterol were detected (Figure 7). As it was found, the negatively correlated regression coefficients used for the prediction of ergosterol were not observed as characteristic peaks in the ergosterol spectrum, while most of the positively correlated regression coefficients (i.e., at 3020, 2921, 2860, 1660, 1353, 1330 and 1043 cm^−1^) corresponded to peaks of the ergosterol spectrum (Figure 7). This revealed a potentially high validity/robustness of the model developed for the prediction of ergosterol as it derived from the spectral regions of the respective standard compound. A direct comparison with previous studies was again not possible since to the best of the authors’ knowledge, it is the first time that a model predicting fungal ergosterol content on the basis of acquired spectroscopic data has been presented. Recently, Shapaval et al. [62] reported a prediction of the total lipid content in oleaginous yeasts by applying high-throughput FTIR spectroscopy and achieved a high R^2^ value for the cross-validation (0.92); however, the R^2^ value achieved during the external validation was 0.67. Moreover, the total sterol content in brown algae was estimated with fairly good accuracy but through the use of a single peak of the FTIR spectrum in conjunction with a previously calibrated standard regression line [63].

## 4. Conclusions

The outcome of the present study indicates that ATR-FTIR could serve as a potential PAT tool for the estimation of glucans and ergosterol contents in *Pleurotus* mushrooms; therefore, it can be exploited as an alternative to the laborious, costly and/or time-consuming analytical techniques/assays used until now. The glucans models could be identified as accurate (low RMSE, low REP and representative regression coefficients) and of a high performance (good *R*^2^), while the RMSE and REP of the ergosterol models can be further improved with the addition of more samples (even though the regression coefficients used in this work for the calibration were extremely accurate). In general, PAT processes and prediction models are dynamic and need to be constantly updated/fed with new entries to sustain/improve their performance. In addition, ATR-FTIR successfully characterized the mushroom samples by detecting differences related to the species or substrates used, and by separating them into groups through a principal component analysis.
